# microRNA 31 functions as an endometrial cancer oncogene by suppressing Hippo tumor suppressor pathway

**DOI:** 10.1186/1476-4598-13-97

**Published:** 2014-04-29

**Authors:** Takashi Mitamura, Hidemichi Watari, Lei Wang, Hiromi Kanno, Makiko Kitagawa, Mohamed Kamel Hassan, Taichi Kimura, Mishie Tanino, Hiroshi Nishihara, Shinya Tanaka, Noriaki Sakuragi

**Affiliations:** 1 Department of Obstetrics and Gynecology, Hokkaido University Graduate School of Medicine, N15, W7, Sapporo, Kita-ku 060-8638, Japan; 2 Department of Cancer Pathology, Hokkaido University Graduate School of Medicine, N15, W7, Sapporo, Kita-ku, Hokkaido 060-8638, Japan; 3 Department of Translational Pathology, Hokkaido University Graduate School of Medicine, Sapporo, Japan; 4 Biotechnology Department, Faculty of Science, Port Said University, Port Fouad, Port Said, Egypt

**Keywords:** Endometrial cancer, microRNA 31, LATS2, cyclin D1, Hippo pathway

## Abstract

**Background:**

We aimed to investigate whether MIR31 is an oncogene in human endometrial cancer and identify the target molecules associated with the malignant phenotype.

**Methods:**

We investigated the growth potentials of MIR31-overexpressing HEC-50B cells *in vitro* and *in vivo*. In order to identify the target molecule of MIR31, a luciferase reporter assay was performed, and the corresponding downstream signaling pathway was examined using immunohistochemistry of human endometrial cancer tissues. We also investigated the MIR31 expression in 34 patients according to the postoperative risk of recurrence.

**Results:**

The overexpression of MIR31 significantly promoted anchorage-independent growth *in vitro* and significantly increased the tumor forming potential *in vivo*. MIR31 significantly suppressed the luciferase activity of mRNA combined with the LATS2 3’-UTR and consequently promoted the translocation of YAP1, a key molecule in the Hippo pathway, into the nucleus. Meanwhile, the nuclear localization of YAP1 increased the transcription of CCND1. Furthermore, the expression levels of MIR31 were significantly increased (10.7-fold) in the patients (n = 27) with a high risk of recurrence compared to that observed in the low-risk patients (n = 7), and this higher expression correlated with a poor survival.

**Conclusions:**

MIR31 functions as an oncogene in endometrial cancer by repressing the Hippo pathway. MIR31 is a potential new molecular marker for predicting the risk of recurrence and prognosis of endometrial cancer.

## Background

Endometrial cancer (EC) is the most common malignancy of the female reproductive tract, the annual incidence of which has been estimated to be 10–20 per 100,000 women [1]. Current therapy for EC includes surgery with adjuvant radiation or chemotherapy [2]. The risk of postoperative recurrence is determined based on several factors, such as the surgical stage [3], differentiation [4], lymph node metastasis and lymphovascular space invasion [5]. The 5-year survival rate for FIGO stage I lesions without grade 3 tumor differentiation, myometrial invasion > 50%, cervical involvement and an adenosquamous histology exceeds 90% [6]. However, the 5-year survival rate of patients with stage III and IV disease is dramatically decreased, ranging from 42% [7] to 79% [8]. EC can be divided into two major categories based on clinicopathologic and molecular genetic features. For example, low-grade carcinomas with PTEN mutations associated with endometrial hyperplasia and estrogenic stimulation, including mucinous or low-grade endometrioid tumors with squamous differentiation, are called type I carcinomas. In contrast, high-grade carcinomas with p53 mutations, such as serous carcinomas and clear cell carcinomas, are referred to as type II cancers [9]. It is important to identify new molecular mechanisms underlying the process of endometrial carcinogenesis and discover molecular targets and novel drugs for improving survival.

microRNAs (MIRs) are endogenous non-coding RNAs of 18 to 25 nucleotides in length that play important roles in regulating the gene expression. The mature forms of MIRs silence the gene expression by binding to the 3’-untranslated region (UTR) of target mRNAs and initiate the translational repression and/or cleavage of cognate mRNAs [10]. MIRs have frequently been implicated in carcinogenesis [11-14]. In the setting of EC, MIR152 [15], MIR194 [16], MIR34b [17], MIR204 [18], MIR145 [19] and MIR129-2 [20] have been reported to be tumor suppressor genes, and MIR125b [21] has been reported to be an oncogene (oncomir). Furthermore, MIR31 has been reported to be an oncomir in various human cancers, including colorectal [22], esophageal [23], lung [24], oral [25] and head and neck [26] cancer, and a tumor suppressor gene in breast [27] and gastric [28] cancers and malignant mesothelioma [29]. However, little is known about the biological functions of MIR31 in EC.

The Hippo pathway is crucial in regulating the size of organs, and its dysregulation contributes to tumorigenesis [30]. Recently, it was reported that deregulation of the Hippo pathway occurs at a high frequency in a broad range of human cancers, including lung [24], hepatocellular [31], colon [32] and prostate cancer [33], and is often correlated with a poor patient prognosis. LATS2 represents a core component in the kinase cascade of the mammalian Hippo pathway. Interestingly, it has been reported that the Hippo pathway is required for anoikis and that the LATS2 expression levels are significantly downregulated in patients with metastatic prostate cancer [33].

In this study, we aimed to investigate whether MIR31 is an oncomir in human EC and identify the direct target associated with the malignant phenotype of EC.

## Results

### MIR31 is correlated with enhanced colony formation of EC cell lines

In order to investigate whether the MIR31 expression is correlated with the tumorigenesis of EC, we performed colony formation assays. We confirmed the MIR31 expression in three EC cell-lines, HEC-50B, HEC-1A and HEC-108, using qRT-PCR and found that the MIR31 levels were lowest in the HEC-108 cells, followed by HEC-1A and HEC-50B cells (Additional file 1: Figure S1, Lanes 1, 2 and 3). The colony number was increased in the same order as the MIR31 expression under two different serum concentrations (Additional file 2: Figure S2).

### The overexpression of MIR31 enhances tumorigenesis in vitro and in vivo

**Figure 1:**
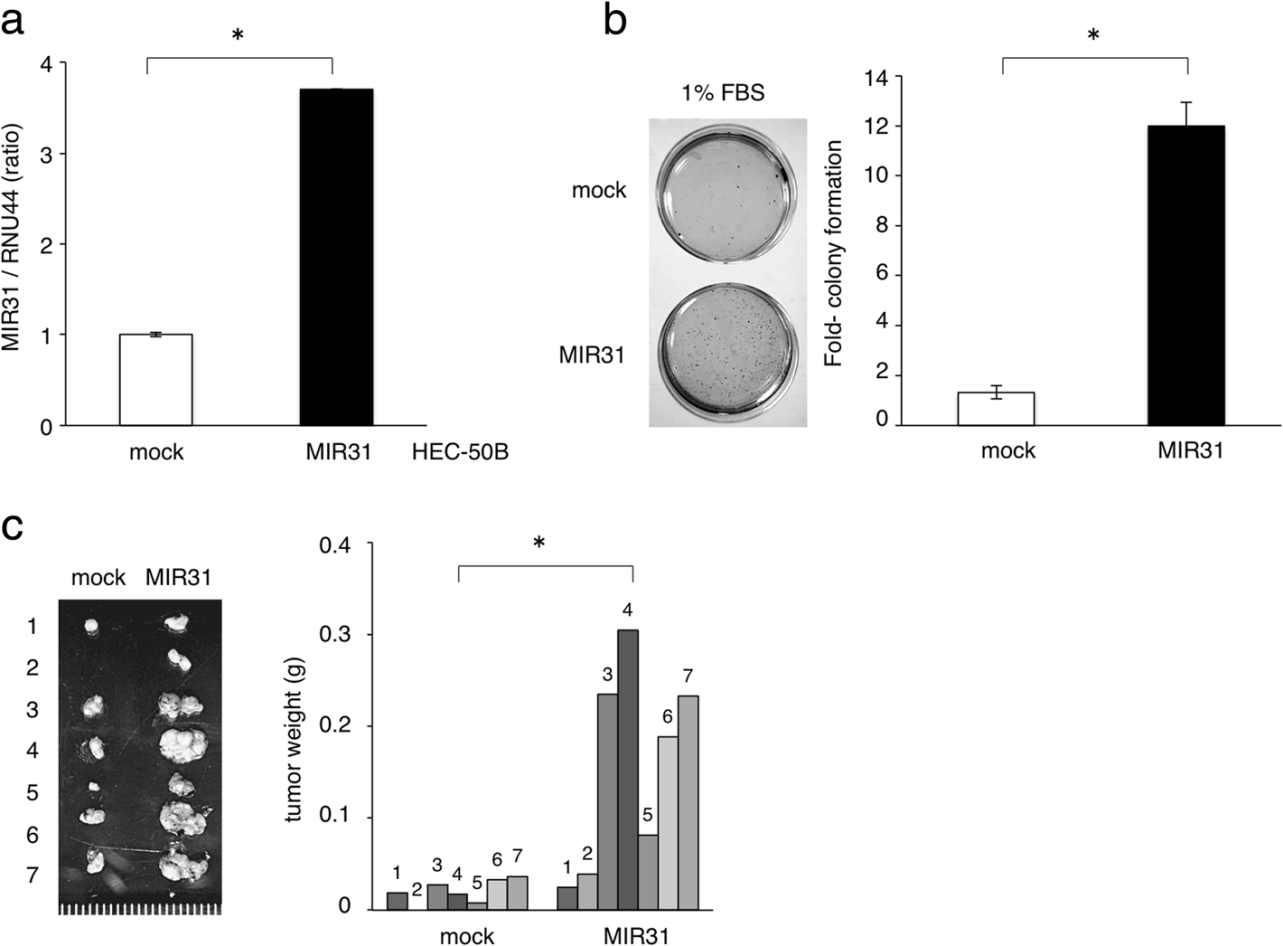
**The overexpression of MIR31 enhanced tumorigenesis *in vitro *and *in vivo. *(a)** Establishment of HEC-50B-expressing MIR31 cells. The results of the qRT-PCR analysis of the expression levels of MIR31 are shown in the bar graph. *p < 0.05, unpaired two-tailed Student’s *t*-test. The experiments were performed in triplicate. **(b)** Colony formation assay with 1% FBS. Representative stained colonies are displayed in the left panel. *p < 0.05, unpaired two-tailed Student’s *t*-test. The experiments were performed in triplicate. **(c)** Subcutaneous tumors in the seven nude mice are displayed in the left panel. The weights of tumors are shown in the right bar graph. *p < 0.05, paired two-tailed Student’s *t*-test.

We established HEC-50B cells overexpressing MIR31 by introducing precursor-MIR31 using lentivirus vectors because the MIR31 expression level of HEC-50B was modest among the several adenocarcinoma cell lines analyzed (Additional file 1: Figure S1) and lentivirus vectors can be efficiently transfected into this cell line (HEC-50B mock and MIR31). The presence of a mature-MIR31 expression was confirmed using qRT-PCR (Figure 1a).

Although MIR31 overexpression did not affect *in vitro* cell proliferation under the standard culture conditions (data not shown), it significantly promoted colony formation under serum starvation (Figure 1b). Additionally, an MIR31-specific inhibitor significantly restrained colony formation (Additional file 3: Figure S3). The MIR31-mediated tumorigenic effects were confirmed in an *in vivo* model. A significant increase in tumor weight was observed in the HEC-50B cells with MIR31 overexpression compared with that noted in the controls in the nude mice subcutaneous tumor model (Figure 1c). These findings demonstrate that MIR31 induces a more aggressive phenotype of EC.

### MIR31 reduces the protein levels of LATS2 by inhibiting translation

**Figure 2:**
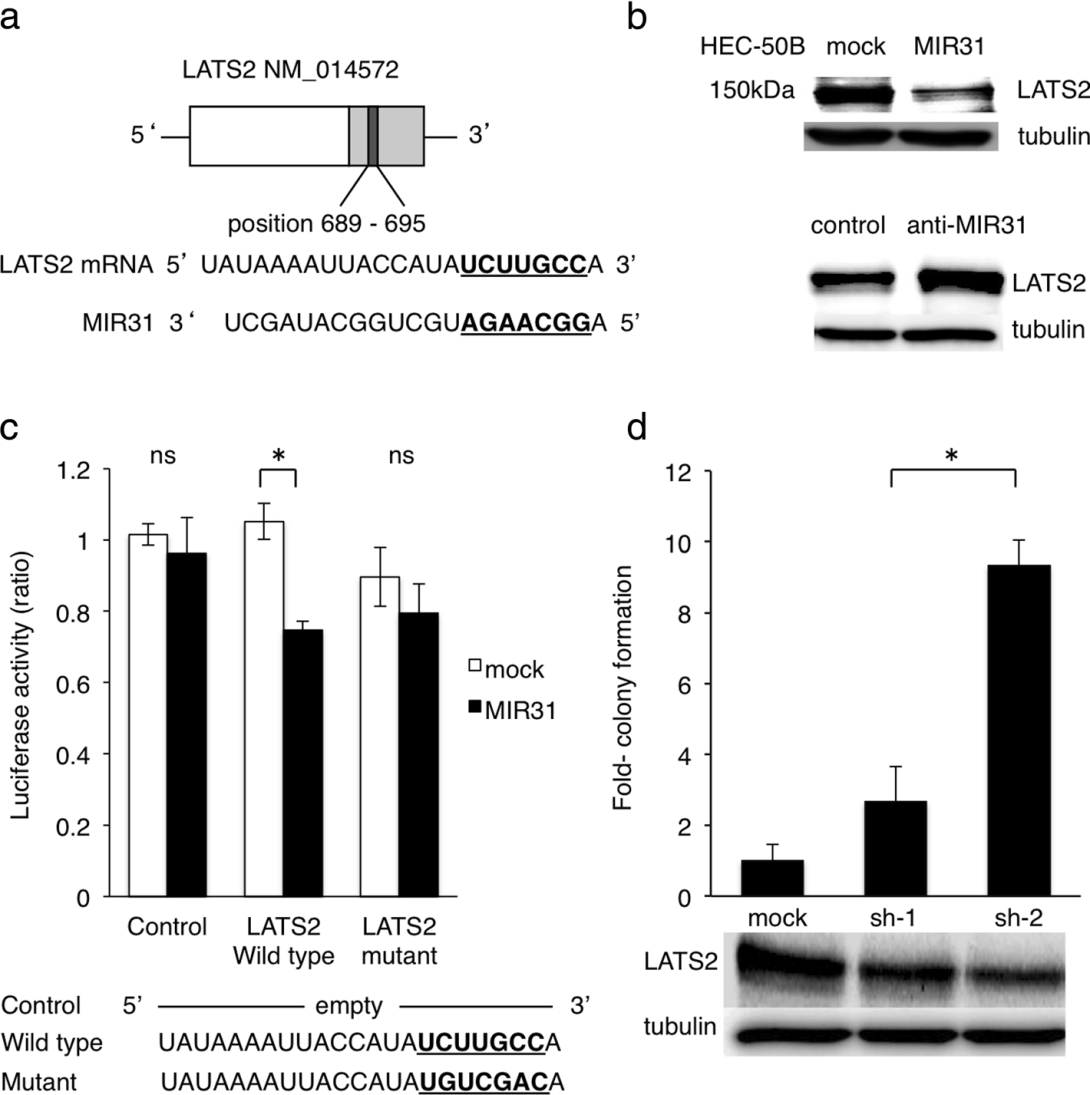
**MIR31 regulates the LATS2 expression by inhibiting translation. (a)** The potential binding site for MIR31 in 3’UTR of LATS2 mRNA. **(b)** The expression level of LATS2 in the mock and MIR31-overexpressing cells (top). The LATS2 expression was increased by the MIR31-specific inhibitor (bottom). The results of immunoblotting for LATS2 and α-tubulin are shown. **(c)** The luciferase activity after transfection of the indicated 3’-UTR-driven reporter constructs. Reporter plasmids containing no oligonucleotides as a Control, the wild-type 3’UTR region of LATS2 as a Wild type and the mutant 3’UTR region as a Mutant. *p < 0.05, unpaired two-tailed Student’s *t*-test. **(d)** Colony formation assay (bar graph) and immunoblotting for LATS2 and α-tubulin following shRNA transfection (bottom panel). *p < 0.05, unpaired two-tailed Student’s *t*-test. All experiments were performed in triplicate.

In order to elucidate the mechanisms by which MIR31 promotes tumorigenesis, *in silico* prediction models were employed to identify the target mRNAs of MIR31 [10]. Among several candidates, we focused on LATS2 because it is a known tumor suppressor gene that has been previously reported to be a direct target of MIR31 [24,34]. One potential binding site for MIR31 was found in the 3’-UTR region of LATS2 mRNA (Figure 2a).

To confirm that LATS2 is a target of MIR31 in HEC-50B cells, the protein levels of LATS2 were analyzed in HEC-50B cells overexpressing MIR31. We found that LATS2 was downregulated in the MIR31-overexpressing cells, whereas LATS2 was increased by the MIR31-specific inhibitor compared with that observed in the control cells in a Western blot analysis (Figure 2b). We next performed a luciferase reporter assay to assess whether MIR31 inhibits the translation of LATS2. The detection of a normalized luciferase activity revealed that MIR31 significantly suppressed the activity of luciferase combined with wild-type LATS2 3’-UTR in the HEC-50B MIR31 cells, whereas no differences were observed following treatment with the control luciferase and LATS2 3’-UTR possessing a mutation in the putative MIR31-binding site (Figure 2c). As no significant differences in the LATS2 mRNA levels were observed between the HEC-50B control and MIR31-overexpressing cells (Additional file 4: Figure S4), MIR31 does not appear to degrade LATS2 mRNA. These results suggest that MIR31 directly binds to LATS2 mRNA and regulates the LATS2 protein expression via translational inhibition.

### Downregulation of LATS2 contributes to tumorigenesis in HEC-50B cells

In order to investigate whether the downregulation of LATS2 is responsible for the enhanced colony-forming ability of HEC-50B cells, the expression of LATS2 was suppressed by two different short hairpin RNAs (shRNAs) (Figure 2d bottom), and the treated cells were evaluated for tumorigenesis using a colony formation assay under low serum concentrations. After 12 weeks of severe starvation (incubation with 1% fetal bovine serum (FBS)), increased colony formation was clearly observed in the cells with LATS2 suppression, whereas treatment with nonspecific shRNA did not affect colony formation (Figure 2d top). We observed the same findings following treatment with 5% FBS for four weeks (Additional file 5: Figure S5). These results suggest that the suppression of the LATS2 expression induced by MIR31 contributes to enhanced tumorigenesis.

### MIR31 promotes the transcription of cyclin D1 (CCND1) via dysregulation of the Hippo signaling pathway

**Figure 3:**
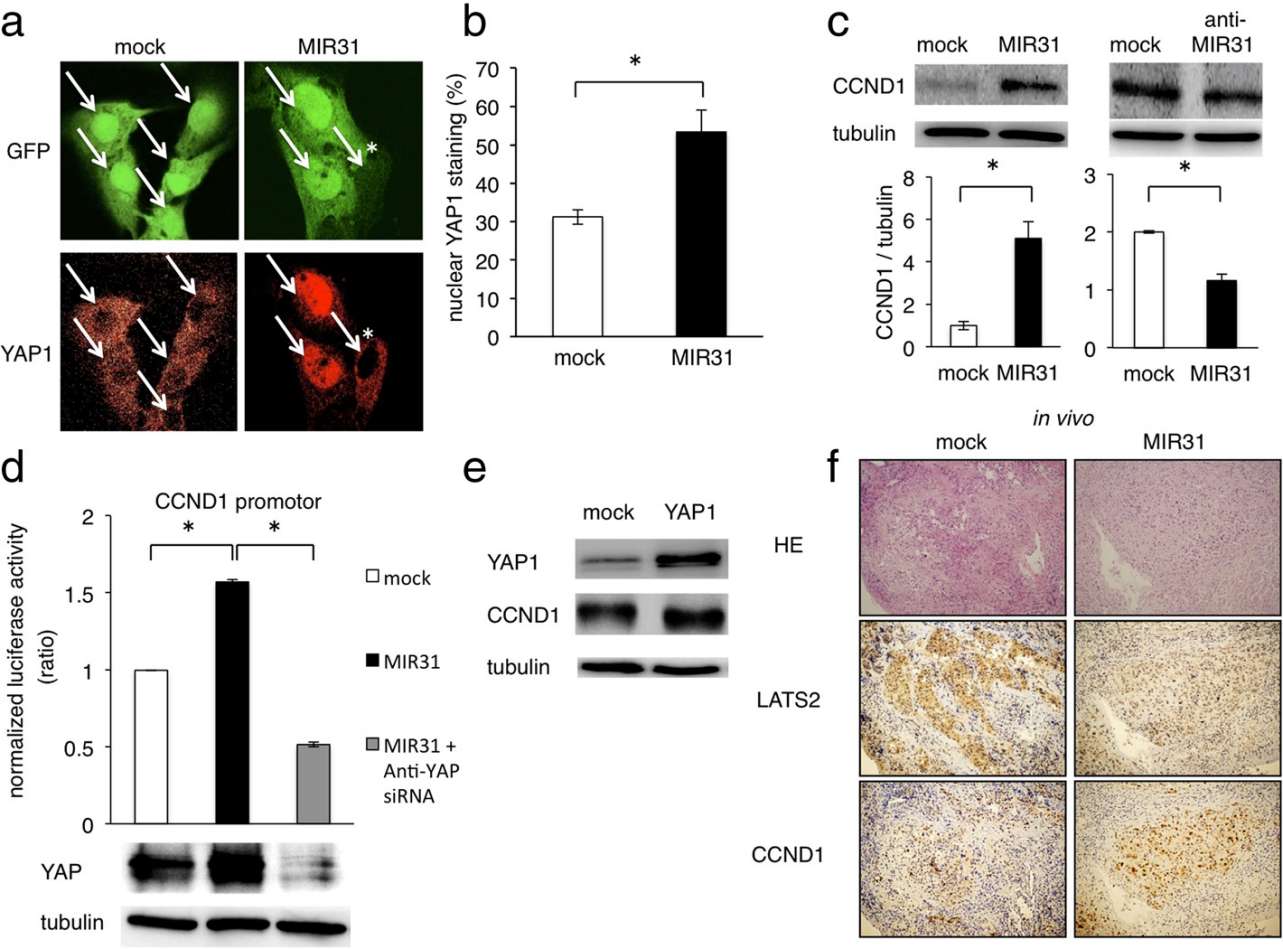
**MIR31 promotes the translocation of YAP1 into the nucleus and promotes the transcription of CCND1. (a)** Representative cells of immunofluorescence for GFP and YAP1, x600. The nuclei are indicated by white arrows. *The translocation of YAP1 into the nucleus was not observed in the cells unsuccessfully transfected with pre-MIR31. The cells were cultured under standard conditions with 5% fetal bovine serum. **(b)** Ratio of nuclear YAP1 staining. *p < 0.05, unpaired two-tailed Student’s *t*-test. **(c)** The CCND1 levels were increased by MIR31 overexpression and the CCND1 levels were decreased by the MIR31-specific inhibitor on immunoblotting for CCND1 and α-tubulin. The ratio of CCND1/α-tubulin is shown in the bar graph. **(d)** The CCND1 levels were increased by YAP1 overexpression. Immunoblotting for YAP1, CCND1 and α-tubulin. **(e)** The luciferase activity after transfection of the reporter constructs containing the LATS2 promotor region normalized to the GAPDH promotor region (top). Immunoblotting for YAP and α-tubulin following siRNA transfection (bottom). Mock and MIR31 cells were transfected with non-targeting siRNA. *p < 0.05, unpaired two-tailed Student’s *t*-test. **(f)** Correlation between the MIR31 expression and results of the immunohistochemical analysis of LATS2 and CCND1 *in vivo*. Representative results are shown in micrographs, x100. All experiments were performed in triplicate.

The Hippo tumor suppressor pathway regulates several cellular functions, including proliferation, survival and metastasis. In the Hippo pathway, the transcriptional coactivator YAP1 translocates into the nucleus, where it promotes the transcription of several target genes associated with proliferation and anti-apoptosis. LATS2 is the key molecule in this pathway and promotes the phosphorylation of YAP1. The phosphorylation of YAP1 by LATS2 inhibits the translocation of YAP1 into the nucleus and thus prevents the transcription of YAP1 target genes [30]. Therefore, when LATS2 is suppressed by MIR31, it is expected that the translocation of YAP1 into the nucleus would be promoted. In order to confirm the YAP1 translocation, we performed immunofluorescence. The cells successfully transfected with control or precursor MIR31 vectors expressed Green Fluorescent Proteins (Figure 3a top), and YAP1 was stained with Cy5 (Figure 3a bottom). As expected, we found that the nuclear translocation of YAP1 frequently occurred in the MIR31-overexpressing cells compared with that observed in the control cells (Figure 3b). In addition, YAP phosphorylation was either not different or slightly increased in the HEC-50B MIR31 cells compared with that observed in the HEC-50B cells (not significant, Additional file 6: Figure S6). These results suggest that the translocation of YAP1 into the nucleus is the most important effect of MIR31.

Because we hypothesized that nuclear YAP1 promotes the transcription of several anti-apoptosis and pro-proliferation genes, we analyzed the expression levels of several proteins, including CCND1, RAS, XIAP, cyclin E1, MYC, KIT, JNK, AKT, FAS, FADD, FASLG and BCL2, in the HEC-50B MIR31-overexpressing and control cells using immunoblotting (Additional file 7: Figure S7). We found the MIR31 overexpression to be associated with increased CCND1, RAS and XIAP expression levels (Figure 3c, Additional file 8: Figure S8a top) and they also decreased in the anti-MIR31-oligonucleotide-induced cells (Figure 3c, Additional file 8: Figure S8a bottom). Because these results strongly suggest that nuclear YAP1, which is increased by the MIR31 expression, promotes the transcription of these targets, we performed luciferase reporter assays in order to investigate the influence of MIR31 on the transcription of CCND1, RAS and XIAP. The detection of a normalized luciferase activity revealed that the MIR31 expression significantly increased the activity of luciferase driven by the CCND1, RAS and XIAP promoters compared with that observed in the control cells (Figure 3d Line 1–2, Additional file 8: Figure S8b). In addition, we focused on the CCND1 expression and investigated the influence of nuclear YAP1 overexpression on the CCND1 expression. HEC-50B cells were transfected with a YAP1 expression vector, the results of which confirmed that nuclear YAP1 was overexpressed on immunofluorescence (Additional file 9: Figure S9). As expected, YAP suppression by anti-YAP siRNA significantly decreased the activity of luciferase driven by the CCND1 promotor, and the expression of CCND1 was increased by YAP1 overexpression (Figure 3d, Lines 2–3, Figure 3e).

We also found a correlation between the MIR31, LATS2 and CCND1 expression *in vivo* (mouse 7 in Figure 1c). The LATS2 expression was increased in the control cell tumors compared with that observed in the MIR31-expressing tumor cells, and the CCND1 levels were increased in the tumors formed from MIR31-expressing cells (Figure 3f).

### Correlations between the MIR31, LATS2 and CCND1 expression in EC

**Figure 4:**
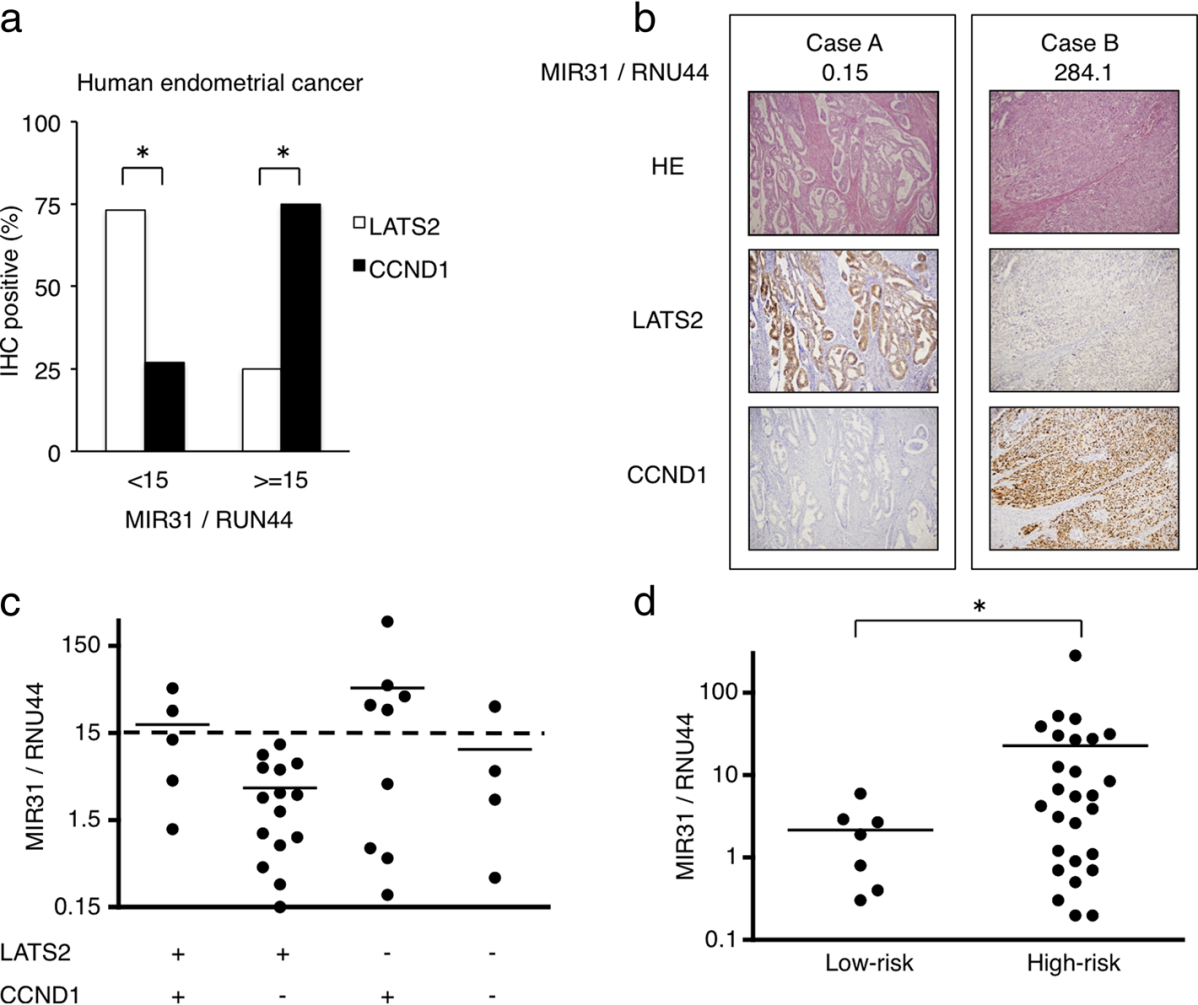
**Correlation between the MIR31 expression and the immunohistochemical detection of LATS2 and CCND1 in human EC cells. (a)** Proportion of patients with positive staining for LATS2 and CCND1 classified into the MIR31 > =15 and MIR31 < 15 groups. *p < 0.05, Chi-square test. **(b)** The MIR31 expression assessed using qRT-PCR with RNU44 as the endogenous control (top) and representative micrographs, x100. **(c)** The MIR31 expression levels in all patients classified according to staining for LATS2 and CCND1 are shown in the scatter diagram. The horizontal solid lines indicate the mean. **(d)** The expression levels of MIR31 in the human EC cells were analyzed using qRT-PCR. The horizontal lines demonstrate the mean. *p < 0.05, Mann-Whitney's U-test.

We compared the MIR31 expression quantified by qRT-PCR and the immunohistochemical expression of LATS2 and CCND1 in 34 EC patients who underwent surgery as their initial treatment (Table 1, Lane 1). When we divided the 34 patients into two groups according to the MIR31 expression (MIR31/RNU44 = 15), the MIR31 expression levels were found to be low in the LATS2-positive (73%) and CCND1-negative (27%) tumors and high in the LATS2-negative (25%) and CCND1-positive (75%) tumors. (Figure 4a; Two representative cases are shown in Figure 4b). The MIR31 expression was lowest in the LATS2-positive and CCND1-negative groups and highest in the LATS2-negative and CCND1-positive groups (Figure 4c).

**Table 1:** Clinical features of human endometrial cancer

	**Total**	**Low-risk**	**High-risk**	**Recurrence**
n (%)	34 (100)	7 (21)	27 (79)	7 (21)
Age, mean (range)	59 (38–78)	58 (38–75)	60 (45–78)	56 (45–67)
FIGO stage, n (%)				
I	15 (44)	7 (100)	8 (30)	1 (14)
II	3 (9)	0 (0)	3 (11)	1 (14)
III	14 (41)	0 (0)	14 (52)	3 (43)
IV	2 (6)	0 (0)	2 (7)	2 (29)
Grade, n (%)				
1	15 (44)	5 (71)	10 (37)	0 (0)
2	13 (38)	2 (29)	11 (41)	6 (86)
3	6 (18)	0 (0)	6 (22)	1 (14)
Lymphovascular space invasion, n (%)				
+	10 (29)	0 (0)	10 (37)	2 (29)
-	24 (71)	7 (100)	17 (63)	5 (71)
MIR31, mean	18.40	2.05	21.90	9.15
(range)	(0.15 - 284.06)	(0.33 - 5.98)	(0.15 - 284.06)	(0.70 - 27.77)

### The MIR31 expression is increased in high-risk human endometrial cancers

**Figure 5:**
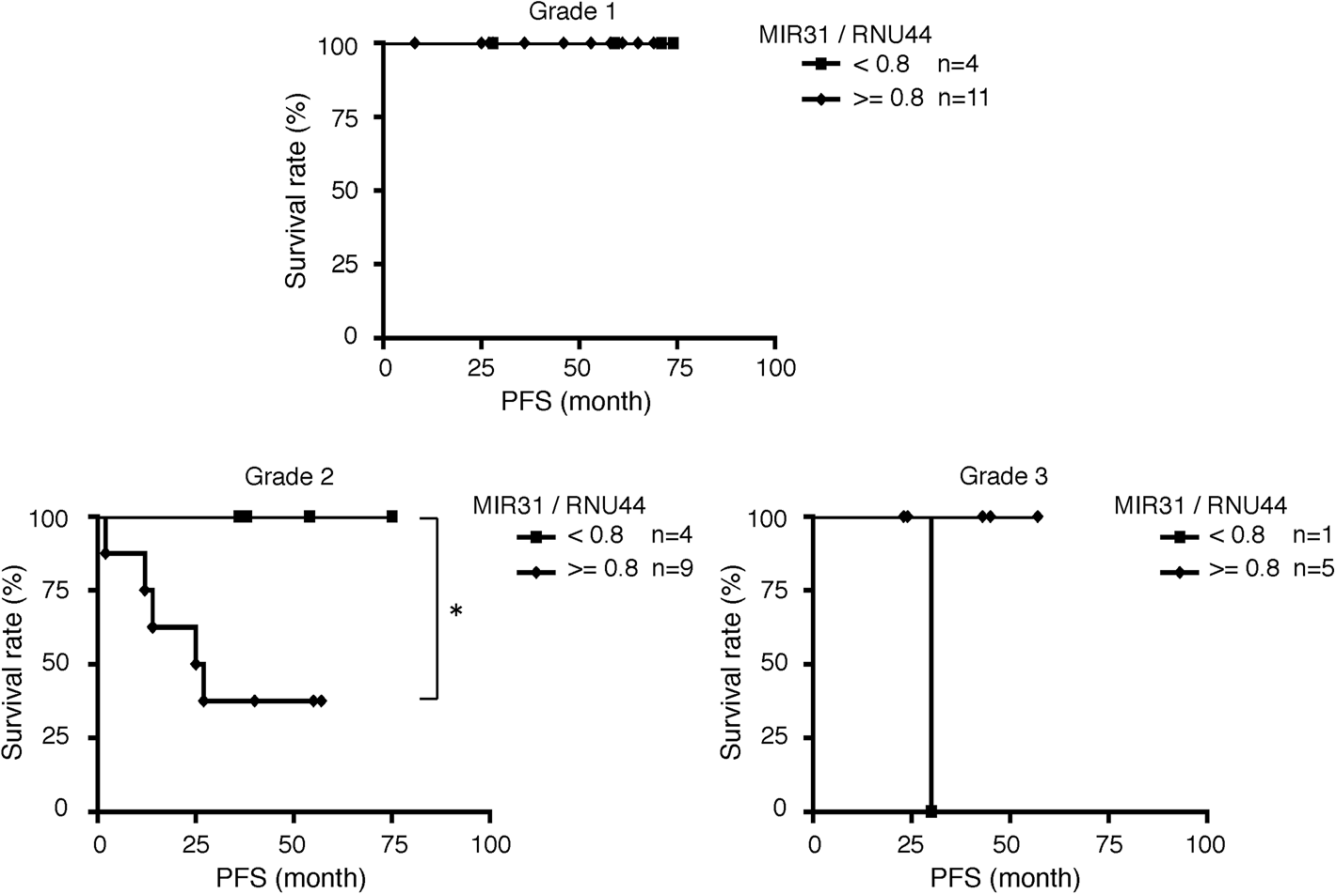
**Prognosis of patients classified into the high and low MIR31 groups.** Kaplan–Meier curves depicting the progression-free survival (PFS). *p < 0.05, log-rank test.

We defined low-risk patients as those who satisfied all of the following criteria: pT1a, pN0, M0, grade1 or 2 without lymphovascular space invasion (Table 1, Lanes 2 and 3). We found that the expression of MIR31 was significantly increased in the high-risk patients (Figure 4d). All patients with recurrent disease were classified as high-risk patients. Since most tumors in the recurrent disease patients were of grade 2 (Table 1, Lane 4), we focused on the prognosis of the 13 patients with grade 2 tumors (Additional file 10: Table S1). As expected, the progression-free survival was significantly worse among the patients with high MIR31 tumors (> = 0.8) than among those with low MIR31 tumors (<0.8) (Figure 5). These results suggest that MIR31 is related to the aggressiveness of EC.

## Discussion

**Figure 6:**
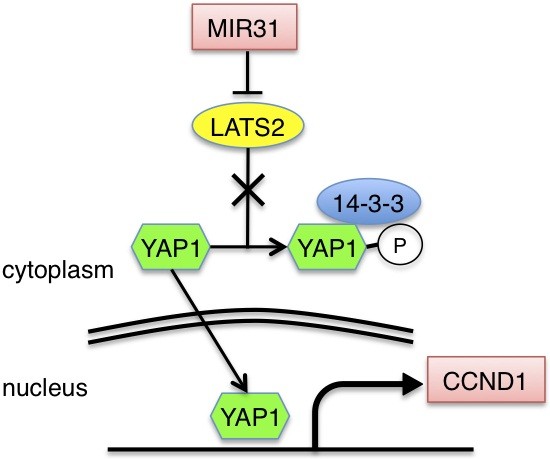
Schematic drawing of the Hippo pathway in human EC.

In this study, we demonstrated that MIR31 functions as an oncomir in EC. MIR31 is an oncomir in several human cancers and a tumor suppressor gene in others. We speculate that MIR31 has a specific function in each type of malignancy, and several mechanisms, including methylation-dependent silencing [35] and local deletion [29], may explain its different roles in different tumor types. However, little is known about the MIR31 status in patients with EC. In previous studies that reported MIR31 to be an oncomir, MIR31 was found to regulate RAS p21 GTPase Activating Protein 1 (RASA1) [22] and RhoBTB1 [36] in colorectal cancer, LATS2 and PP2A regulatory subunit B alpha isoform (PPP2R2A) [24] in lung cancer and factor-inhibiting hypoxia-inducible factor (FIH) [26] in head and neck carcinomas. It is plausible that MIR31 represses the expression of tumor suppressor genes, such as LATS2, to act as an oncomir and indirectly promotes the transcription of genes related to cell cycle control and tumorigenesis (Figure 6).

We herein demonstrated that the Hippo pathway is involved in EC tumorigenesis and correlates with a poor patient prognosis. Although no significant relationships were observed between the immunohistochemical expression of LATS2 and clinical risk factors, including lymph node metastasis, cervical invasion or lymphovascular space invasion, such relationships deserve further investigation in a larger patient cohort. It is reasonable to postulate that the Hippo pathway regulates the CCND1 expression via YAP1 translocation into the nucleus in EC, as the cyclin family is known to be a major target of the Hippo pathway [37-39]. The overexpression of CCND1 may not be an independent factor causing tissue overgrowth, since it is suggested that the overexpression of all known yorkie targets fails to mimic the effect of yorkie itself in driving tissue growth in drosophila [40]. As MIR31 tends to block the cell apoptosis induced by ultraviolet treatment in HEC-50B cells (data not shown), we speculate that other transcriptional targets of YAP1 associated with apoptosis, such as XIAP, may be coordinately regulated with CCND1. The Hippo pathway is known to be related to the p53 activity, for example, LATS2 tumor suppressor augments p53-mediated apoptosis by promoting the nuclear proapoptotic function of ASPP1 [41,42]. On the other hand, a p53 mutation is found in some patients with aggressive histologic subtypes of endometrial cancer [43]. Our findings suggested the existence of a possible connection between MIR31 and p53 mutation in EC which thus induces the aggressiveness of EC. Additionally, the connection between MIR31 and the p53 mutation could therefore explain the reason why MIR31 either promotes or suppresses different cancers.

As mentioned above, recommendations for postoperative adjuvant therapy for EC are based on the risk assessment of recurrence for each individual patient. In this study, we divided 34 EC patients into two groups according to the criteria generally used to determine whether postoperative adjuvant therapy is required and found a strong correlation between the MIR31 expression and these clinical risk factors. These results suggest that MIR31 is potentially a new molecular marker for distinguishing the risk of recurrence combined with histological findings. However, the small sample size of the present study limits the robustness of our findings, and further investigation in a larger patient cohort is necessary.

## Conclusions

In conclusion, we herein demonstrated that MIR31 promotes EC tumorigenesis. MIR31 indirectly promotes the translocation of YAP1 into the nucleus by repressing the tumor suppressor gene LATS2.

## Methods

### Human endometrial tumor tissues

Tumor specimens were obtained from patients with EC treated at Hokkaido University Hospital under institutional review board approval (registration ID: 011–0157). Informed consent was obtained from each subject. Patients treated at Hokkaido University Hospital between 2006 and 2012 were eligible for inclusion. All samples were obtained at the initial surgery. RNA was extracted using the RecoverAll™ Total nucleic Acid Isolation Kit (Ambion, Austin, TX, USA) from formalin-fixed, paraffin-embedded tissues. We set the samples on the slide glass and microscopically recognized the malignantly transformed epithelial lesion, then cored out the epithelial lesion. MIR31 was detected using quantitative real-time PCR (qRT-PCR). All experiments were performed three times, and ratio of the mean MIR31 level relative to the endogenous control RNU44 level was calculated.

### qRT-PCR

Total RNA was extracted using TRIzol Reagent (Invitrogen, Carlsbad, CA, USA). The MIR31 and RNU44 levels were quantified using qRT-PCR with the TaqMan® MicroRNA Reverse Transcription Kit (Applied Biosystems, Foster City, CA, USA) and TaqMan® MicroRNA Assays (Applied Biosystems) according to the manufacturer's instructions. We assessed the RNA expression according to relative quantification using the 2^-ΔΔCt^ method [44] to determine the fold change in the expression.

### Cell lines

The human EC cell lines HEC-50B, HEC-1-A and HEC-108 were obtained from RIKEN BioResource Center (Tsukuba, Japan) and maintained in Dulbecco's Modified Eagle’s Medium (DMEM) with 10% FBS, 2 mM L-glutamine and 100 U/ml of penicillin and streptomycin in a 6-cm dish. SK-OV-3 and OVCAR-3 were obtained from the ATCC (Manassas, VA, USA) and maintained in McCoy’s 5a Medium with 10% FBS, 2 mM L-glutamine and 100 U/ml of penicillin and streptomycin and in Roswell Park Memorial Institute medium 1640 with 20% FBS, 0.01 mg/ml of bovine insulin, 2 mM L-glutamine and 100 U/ml of penicillin and streptomycin.

### Overexpression of MIR31

Precursor-MIR31 was transfected into HEC-50B using the BLOCK-iT™ Lentiviral miR RNAi Expression System (Invitrogen, Carlsbad, CA, USA) following the manufacturer’s protocol, as previously described [45]. After transfection, we performed blasticidin selection at a concentration of 2.5 μg/ml for 10 days.

### Colony formation assay

A total of 1.0 × 10^5^ cells were seeded in a layer of 0.4% noble agar/DMEM/1% FBS/0.5 μg/ml of puromycin or 0.4% noble agar/DMEM/5% FBS over a layer of 0.5% bacto agar/DMEM/1% or 5% FBS in a 6-cm dish. The colonies were stained using 3-[4,5-dimethylthiazol-2-yl]-2,5-diphenyltetrazolium bromide solution (Sigma-Aldrich, St. Louis, MO, USA) and counted.

### Analysis of the tumor-forming potential in vivo

All experiments were conducted in accordance with guidelines authorized by the Animal Research Committee of Hokkaido University. Six-week-old BALB/c nude mice (Clea, Tokyo, Japan) were injected subcutaneously into their flanks with 2 x 10^7^ HEC-50B mock or HEC-50B MIR31 cells bilaterally in 200 μl of normal culture medium. All mice were sacrificed on day 28, and the tumor weight was measured.

### Immunoblotting

SDS–PAGE and immunoblotting were carried out as described elsewhere [45]. Briefly, filters were incubated with rabbit polyclonal antibodies against LATS2, mouse monoclonal antibodies against α-tubulin (1:1,000 dilution, Abcam, Cambridge, UK), rabbit polyclonal antibodies against CCND1 (1:500 dilution, Santa Cruz Biotechnology, Dallas, TX, USA), rabbit polyclonal antibodies against YAP1 and phospho-YAP (Ser127) (1:1,000 dilution, Cell Signaling Technology, Danvers, MA, USA), mouse monoclonal antibodies against RAS (1:1,000 dilution, BD Biosciences, San Jose, CA, USA) and rabbit polyclonal antibodies against XIAP (1:100 dilution, Abnova, Taipei City, Taiwan).

### Luciferase reporter assay

To investigate the translation of LATS2, luciferase reporter assay was carried out as described elsewhere [45]. The wild-type (NM_014572) or mutant LATS2 3’-UTR sequence was inserted downstream of the firefly luciferase reporter gene, which was controlled by the SV40 enhancer for expression in mammalian cells, whereas no oligonucleotides were inserted in the control vector (Genecopoeia, Rockville, MD, USA). Renilla luciferase was used as a tracking indicator for successful transfection. In order to investigate the transcription of CCND1, HRAS, KRAS and XIAP, luciferase reporter constructs for the promoters of these molecules and a positive control of glyceraldehyde-3-phosphate dehydrogenase (GAPDH) were obtained (SwitchGear Genomics, Menlo Park, CA, USA). The luciferase activity was measured using LightSwitch Assay Reagent (SwitchGear Genomics) according to the manufacturer's instructions. Briefly, 1.0 to 1.5 × 10^4^ cells were seeded in white 96–well plates on day 1 and transfected with reporter constructs on day 2 using FuGENE HD (Promega). The luciferase activity was measured using assay reagent 48 hours after transfection.

### RNA interference for LATS2 and YAP1

Two shRNA lentiviruses against LATS2 (Sigma-Aldrich) and non-targeting shRNA (Sigma-Aldrich) were transfected into HEC-50B cells in 48-well plates according to the manufacturer's instructions. The multiplicity of infection (MOI, number of transducing lentiviral particles per cell) was 5. We performed puromycin selection at a concentration of 0.5 μg/ml for 10 days. siRNAs against YAP and non-targeting siRNA (Santa Cruz Biotechnology) were transfected using HiPerFect transfection reagent (Qiagen, Tokyo, Japan) in 12-well plates, and the cells were harvested after 48 hours.

### Reverse transcription-PCR

Reverse transcription-PCR was carried out as described elsewhere [45]. The primers used for the expression analysis were as follows: pre- MIR31 - forward, 5'-GGAGAGGAGGCAAGATGCTG-3'; pre- MIR31 - reverse, '-GGAAAGATGGCAATATGTTG-3': GAPDH - forward, 5'-CTCATGACCACAGTCCATGC-3': GAPDH - reverse, 5'-TTACTCCTTGGAGGCCATGT-3': LATS2 - forward, 5'-TAGAGCAGAGGGCGCGGAAG -3': LATS2 - reverse, 5'- CCAACACTCCACCAGTCACAGA-3'.

### Immunofluorescence

Cells were grown on 35-mm glass-based dishes (Asahi Glass, Tokyo, Japan), fixed with 3% paraformaldehyde and permeabilized with 0.1% Triton X-100/PBS before blocking with 1% BSA. The cells were incubated with rabbit polyclonal antibodies against YAP1 (1:500 dilution, Cell Signaling Technology) and secondary antibodies, including goat antibodies to rabbit coupled to Alexa 594 (1:250 dilution, Invitrogen). All samples were examined using laser-scanning confocal microscopy (Fluoview™, Olympus, Tokyo, Japan).

### MIR inhibitor

A total of 200 nM of miRIDIAN microRNA Hairpin Inhibitor and its negative control (Thermo Scientific Dharmacon, Lafayette, CO, USA) were employed to transiently inhibit MIR31 and transfected 48 hours prior to seeding with Oligofectamine (Invitrogen).

### Overexpression of Yes-associated protein 1 (YAP1)

The human YAP1 expression vector, p2xFLAG-YAP1 and negative control were kindly provided by Dr. Sudol [46]. HEC-50B cells at 80% confluence in 6-well plates were transfected with 4 μg of YAP1 vector using FuGENE HD (Promega).

### Immunohistochemistry (IHC)

Formalin-fixed, paraffin-embedded tissues were used to detect the LATS2 and CCND1 expression. The sections were incubated with anti-LATS2 rabbit polyclonal antibodies (Abcam, Cambridge, UK) at 1:300 dilution and anti-CCND1 rabbit monoclonal antibodies (Dako, Glostrup, Denmark) at 1:50 dilution. A semi-quantitative scoring system was used to evaluate the intensity of staining: low (proportion: 0 to 50%, intensity: no staining to weak) and high (proportion: more than 50%, intensity: intermediate to strong).

### Statistical analysis

The data are presented as the mean ± SEM. The unpaired two-tailed Student’s *t*-test, Mann-Whitney's U-test and Chi-square test were used for comparisons, with a p value of < 0.05 considered to be significant (*).

## Competing interests

The authors declare that they have no competing interests.

## Authors’ contributions

TM, HW, HN, ST and NS designed the experiment, interpreted the data and prepared the manuscript. TM, LW, HK, MK, MKH, TK, MT conducted the experiment, collected the data and helped to prepare the manuscript. All authors read and approved the final manuscript.

## Supplementary Material

Additional file 1: Figure S1 qRT-PCR analysis of the MIR31 expression in five adenocarcinoma cell lines of the female genital tract. Click here for file

Additional file 2: Figure S2 Colony formation assay. *p < 0.05, unpaired two-tailed Student’s *t*-test compared with HEC-108. Click here for file

Additional file 3: Figure S3 Colony formation assay, four weeks. *p < 0.05, unpaired two-tailed Student’s *t*-test. Click here for file

Additional file 4: Figure S4 Detection of LATS2 and GAPDH mRNA using RT–PCR. Click here for file

Additional file 5: Figure S5 Representative results of the colony formation assays with 5% FBS for four weeks. Click here for file

Additional file 6: Figure S6 MIR31 is not involved in YAP phosphorylation. Immunoblotting for phospho-YAP and α-tubulin. Click here for file

Additional file 7: Figure S7 Immunoblotting for putative targets of YAP1. Click here for file

Additional file 8: Figure S8 (a) The expression levels of RAS and XIAP in the mock and MIR31-overexpressing cells (top). The RAS and XIAP levels were decreased by the MIR31-specific inhibitor (bottom). Results of immunoblotting for RAS, XIAP and α-tubulin. (b) The luciferase activity after transfection of the reporter constructs containing the HRAS, KRAS and XIAP promotor region normalized to the GAPDH promotor region. *p < 0.05, unpaired two-tailed Student’s *t*-test. Click here for file

Additional file 9: Figure S9 Representative immunofluorescence analysis of YAP1, x600. Click here for file

Additional file 10: Table S1 Correlation between the MIR31 expression and risk of postoperative recurrence in the patients with grade 2 tumors. Click here for file
