# Correction: microRNA 31 functions as an endometrial cancer oncogene by suppressing Hippo tumor suppressor pathway

**DOI:** 10.1186/1476-4598-13-140

**Published:** 2014-06-04

**Authors:** Takashi Mitamura, Hidemichi Watari, Lei Wang, Hiromi Kanno, Masaya Miyazaki, Makiko Kitagawa, Mohamed Kamel Hassan, Peixin Dong, Taichi Kimura, Mishie Tanino, Hiroshi Nishihara, Shinya Tanaka, Noriaki Sakuragi

**Affiliations:** 1 Department of Obstetrics and Gynecology, Hokkaido University Graduate School of Medicine, Sapporo, Japan; 2 Department of Cancer Pathology, Hokkaido University Graduate School of Medicine, Sapporo, Japan; 3 Department of Translational Pathology, Hokkaido University Graduate School of Medicine, Sapporo, Japan; 4 Biotechnology Program, Zoology Department, Faculty of Science, PS U, Port Said University, Port Fouad, Port Said, Egypt; 5 Department of Women’s Health Educational System, Hokkaido University School of Medicine, Hokkaido University, Sapporo, Japan

## Correction

After publication of this work [1], we noted that we inadvertently failed to include the complete list of all coauthors. The full list of authors has now been added and the Authors’ Contributions and Competing Interests sections modified accordingly.

## Competing interests

The authors declare that they have no competing interests.

## Authors’ contributions

TM, HW, HN, ST and NS designed the experiment, interpreted the data and prepared the manuscript. TM, LW, HK, MM, MK, MKH, PD, TK, MT conducted the experiment, collected the data and helped to prepare the manuscript. All authors read and approved the final manuscript.
