# Supplementary figures and images for: microRNA 31 functions as an endometrial cancer oncogene by suppressing Hippo tumor suppressor pathway
Source: Mol Cancer. 2014 Apr 29;13:97. doi: 10.1186/1476-4598-13-97 (PMC4067122; doi:10.1186/1476-4598-13-97)

Supplementary Figure S1

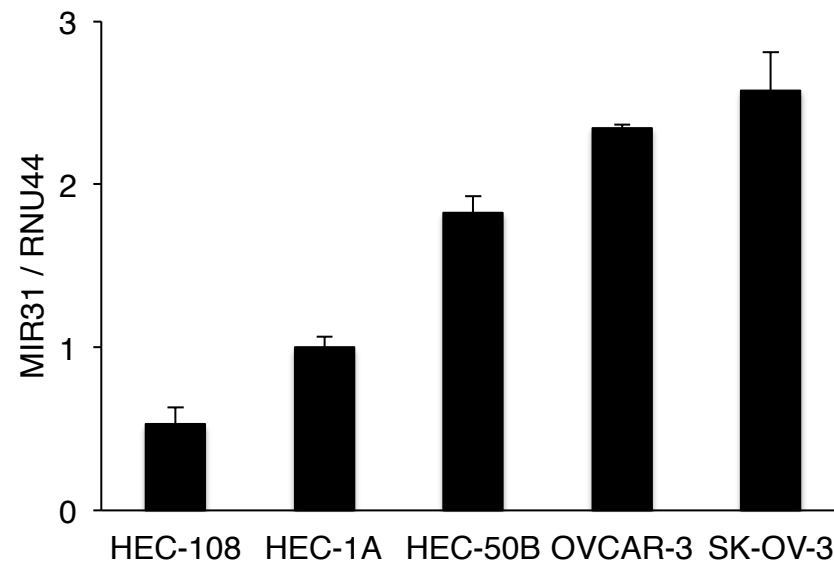

Supplement: Additional file 1: Figure S1 — qRT-PCR analysis of the MIR31 expression in five adenocarcinoma cell lines of the female genital tract. [file 1476-4598-13-97-S1.pdf]

Supplementary Figure S2

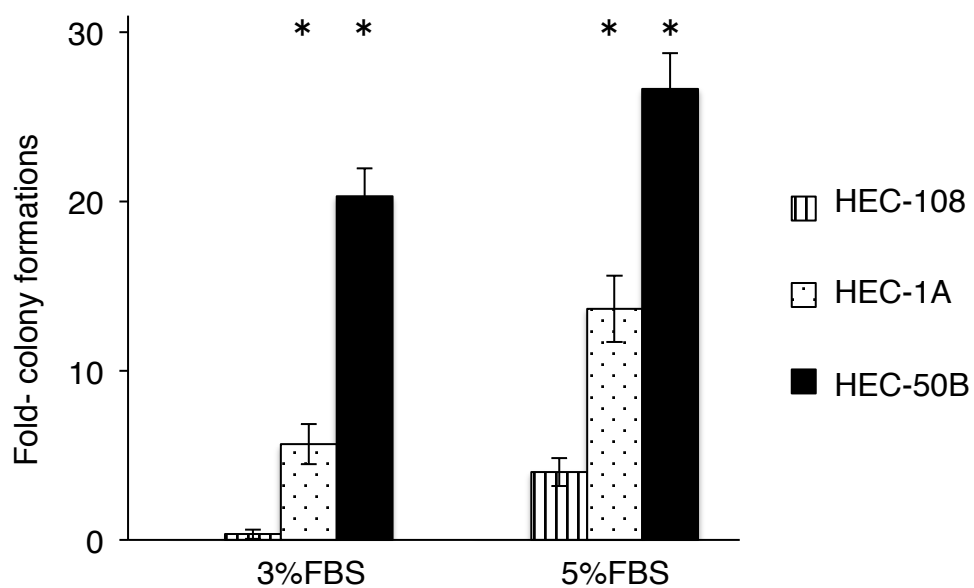

Supplement: Additional file 2: Figure S2 — Colony formation assay. *p < 0.05, unpaired two-tailed Student’s t-test compared with HEC-108. [file 1476-4598-13-97-S2.pdf]

Supplementary Figure S3

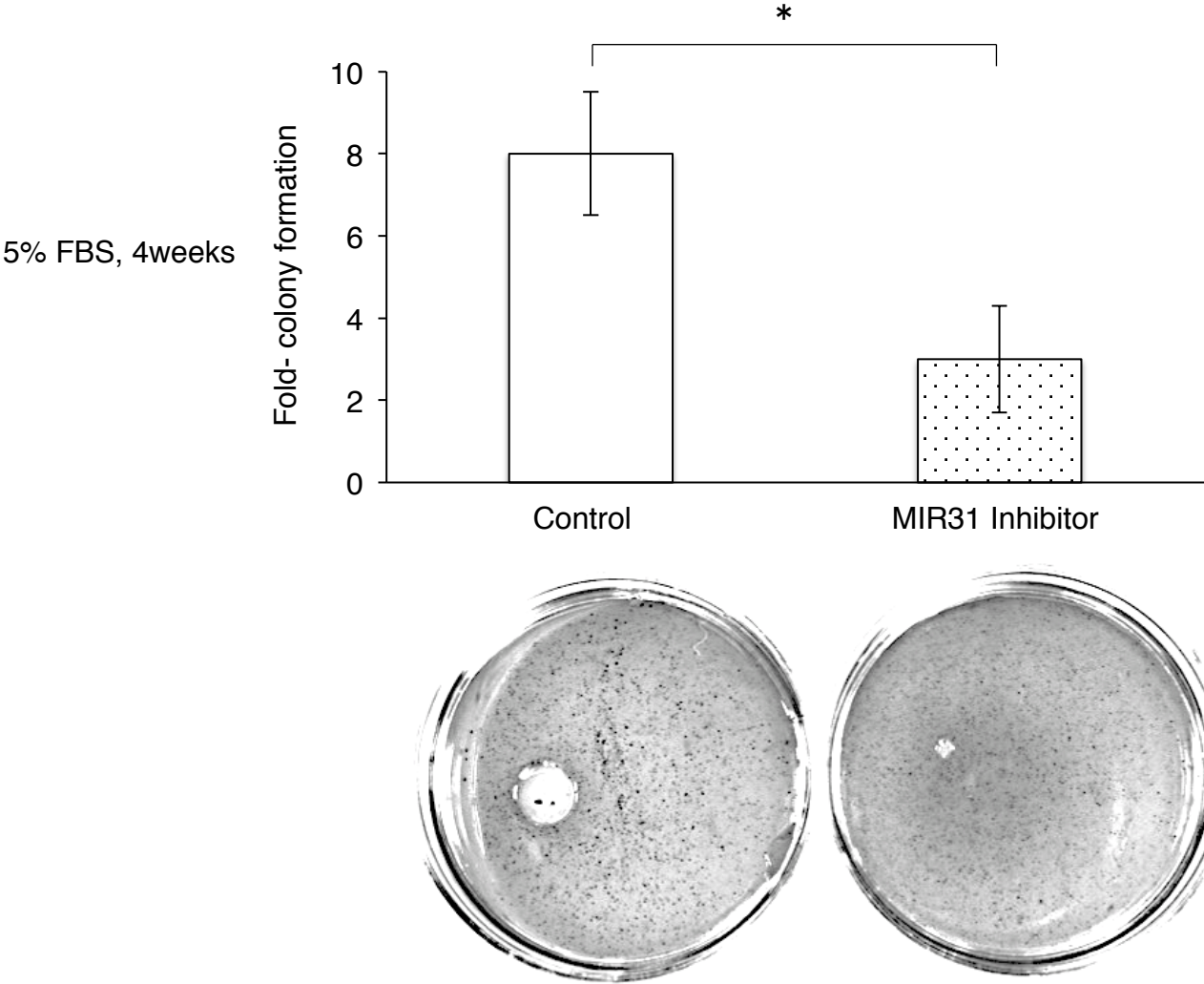

Supplement: Additional file 3: Figure S3 — Colony formation assay, four weeks. *p < 0.05, unpaired two-tailed Student’s t-test. [file 1476-4598-13-97-S3.pdf]

Supplementary Figure S4

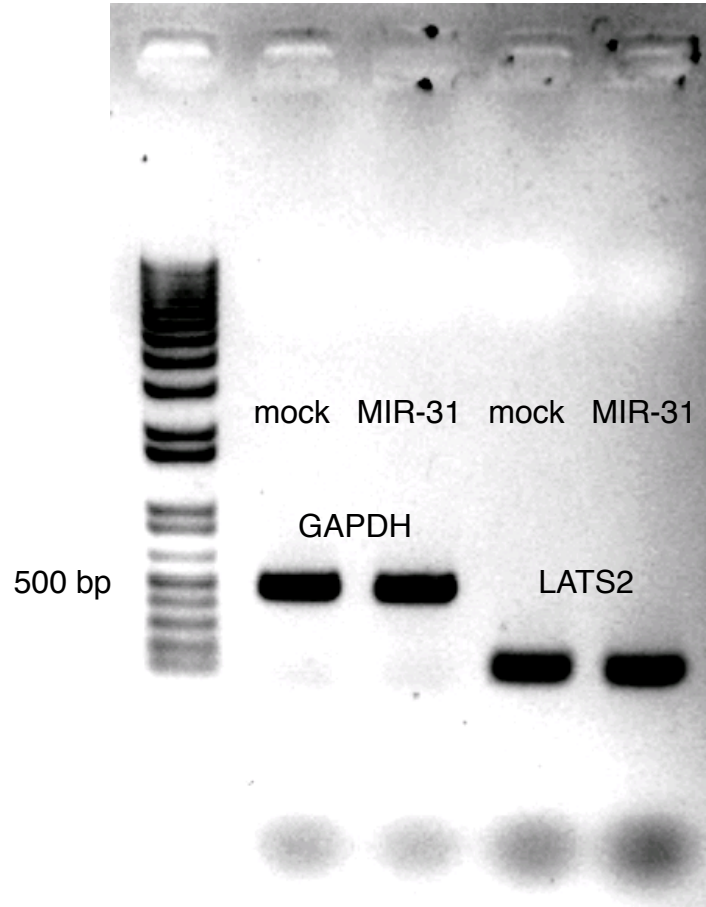

Supplement: Additional file 4: Figure S4 — Detection of LATS2 and GAPDH mRNA using RT–PCR. [file 1476-4598-13-97-S4.pdf]

## Supplementary Figure S5

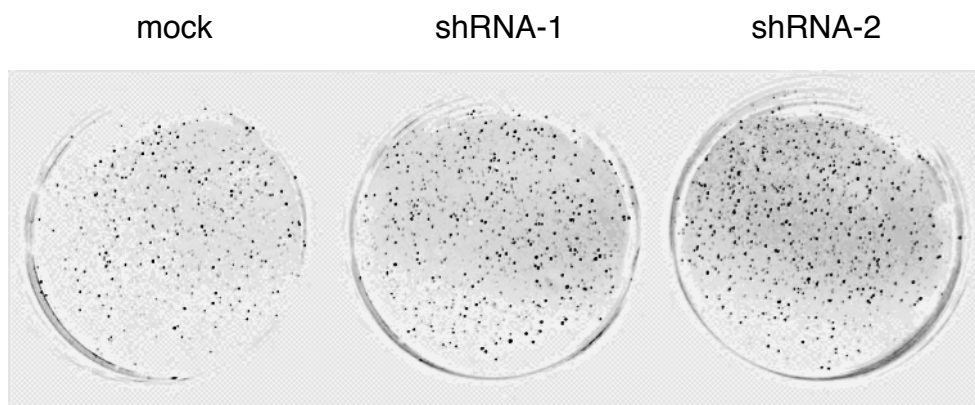

Supplement: Additional file 5: Figure S5 — Representative results of the colony formation assays with 5% FBS for four weeks. [file 1476-4598-13-97-S5.pdf]

## Supplementary Figure S6

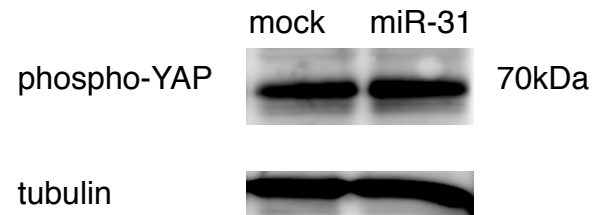

Supplement: Additional file 6: Figure S6 — MIR31 is not involved in YAP phosphorylation. Immunoblotting for phospho-YAP and α-tubulin. [file 1476-4598-13-97-S6.pdf]

## Supplementary Figure S7

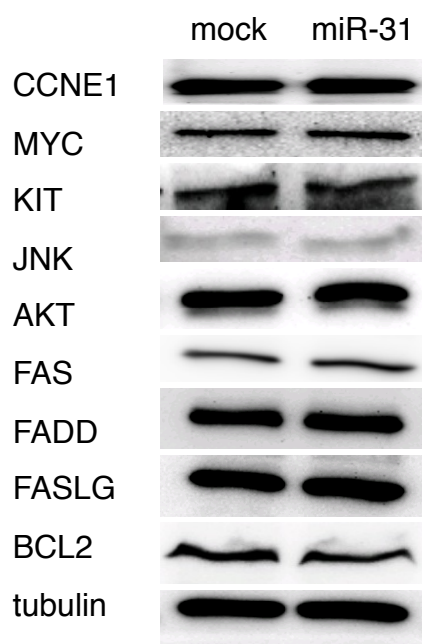

Supplement: Additional file 7: Figure S7 — Immunoblotting for putative targets of YAP1. [file 1476-4598-13-97-S7.pdf]

## Supplementary Figure S8

a

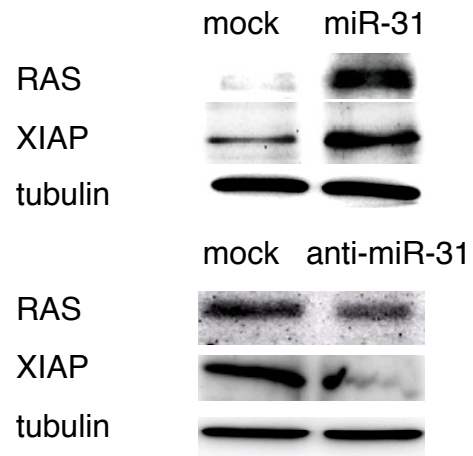

b

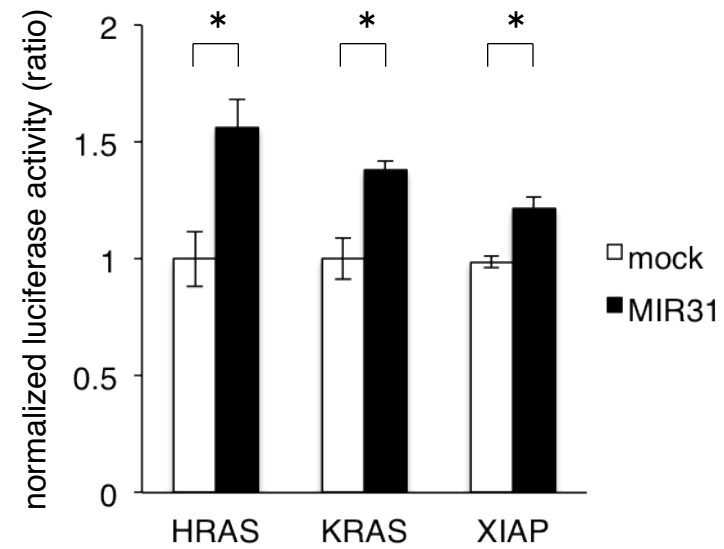

Supplement: Additional file 8: Figure S8 — (a) The expression levels of RAS and XIAP in the mock and MIR31-overexpressing cells (top). The RAS and XIAP levels were decreased by the MIR31-specific inhibitor (bottom). Results of immunoblotting for RAS, XIAP and α-tubulin. (b) The luciferase activity after transfection of the reporter constructs containing the HRAS, KRAS and XIAP promotor region normalized to the GAPDH promotor region. *p < 0.05, unpaired two-tailed Student’s t-test. [file 1476-4598-13-97-S8.pdf]

## Supplementary Figure S9

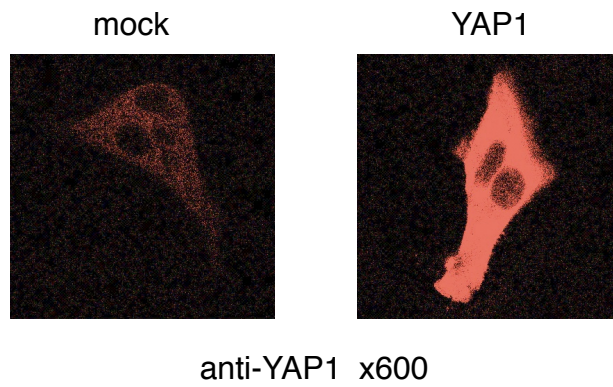

Supplement: Additional file 9: Figure S9 — Representative immunofluorescence analysis of YAP1, x600. [file 1476-4598-13-97-S9.pdf]
