# Supplementary material for: microRNA 31 functions as an endometrial cancer oncogene by suppressing Hippo tumor suppressor pathway
Source: Mol Cancer. 2014 Apr 29;13:97. doi: 10.1186/1476-4598-13-97 (PMC4067122; doi:10.1186/1476-4598-13-97)
Supplement: Additional file 10: Table S1 — Correlation between the MIR31 expression and risk of postoperative recurrence in the patients with grade 2 tumors. [file 1476-4598-13-97-S10.pdf]

Supplementary Table S1

	Low-risk	High-risk	Total
miR-31 low	1	3	4
miR-31 high	1	8	9
Total	2	11	13
